# Self-Supervised Learning Framework toward State-of-the-Art Iris Image Segmentation

**DOI:** 10.3390/s22062133

**Published:** 2022-03-09

**Authors:** Wenny Ramadha Putri, Shen-Hsuan Liu, Muhammad Saqlain Aslam, Yung-Hui Li, Chin-Chen Chang, Jia-Ching Wang

**Affiliations:** 1 Department of Computer Science and Information Engineering, National Center University, Taoyuan 32001, Taiwan; wenny@g.ncu.edu.tw (W.R.P.); 109522071@cc.ncu.edu.tw (S.-H.L.); saqlain@g.ncu.edu.tw (M.S.A.); jcw@csie.ncu.edu.tw (J.-C.W.); 2 AI Research Center, Hon Hai Research Institute, Taipei 114699, Taiwan; yunghui.li@foxconn.com; 3 Department of Information Engineering and Computer Science, Feng Chia University, Taichung 40724, Taiwan

**Keywords:** data augmentation, iris segmentation, generative adversarial network, image semantic segmentation, biometrics

## Abstract

Iris segmentation plays a pivotal role in the iris recognition system. The deep learning technique developed in recent years has gradually been applied to iris recognition techniques. As we all know, applying deep learning techniques requires a large number of data sets with high-quality manual labels. The larger the amount of data, the better the algorithm performs. In this paper, we propose a self-supervised framework utilizing the pix2pix conditional adversarial network for generating unlimited diversified iris images. Then, the generated iris images are used to train the iris segmentation network to achieve state-of-the-art performance. We also propose an algorithm to generate iris masks based on 11 tunable parameters, which can be generated randomly. Such a framework can generate an unlimited amount of photo-realistic training data for down-stream tasks. Experimental results demonstrate that the proposed framework achieved promising results in all commonly used metrics. The proposed framework can be easily generalized to any object segmentation task with a simple fine-tuning of the mask generation algorithm.

## 1. Introduction

Over the past few years, iris recognition has emerged as one of the most suitable and trustworthy biometric modalities among those currently available in the private sector [1,2,3,4]. Automated iris recognition systems, therefore, have been extensively installed in several biometrics applications, including [5], border-crossing control [6,7], citizenship verification [8], digital forensic, and industrial products. Furthermore, iris authentication is profoundly secure because no two irises are identical, even in indistinguishable twins, and the iris is the most precise human identifier apart from Deoxyribonucleic acid (DNA) [9]. Nevertheless, the iris recognition system has now been operating globally, and it represents one of the most developed categories of biometric recognition technology [10]. In addition, it can solve technical obstacles when face recognition is failed or unavailable, peculiarly when the user’s face is covered by masks, especially in the COVID-19 era. The iris recognition framework proposed by Daugman [11,12,13,14] laid the foundation for the entire iris recognition technology. A typical iris recognition system contains the subsequent steps: iris image acquisition, image preprocessing, iris segmentation, feature extraction, and feature matching. Iris segmentation plays an essential role in iris recognition to achieve a high recognition rate. The accurate iris segmentation, combined with the best features and effective recognition schemes, makes the iris recognition system more perfect. However, if the iris segmentation is not accurate, the best feature extraction and recognition algorithms cannot compensate for such defects. As a consequence, the performance of the iris recognition system will drop sharply. Thus, the accuracy of the iris segmentation is enormously significant [15]. Over the past decades, with the rapid development of deep learning, a vast number of investigations employing CNNs have been introduced for iris segmentation [1,16,17,18,19,20], iris bounding box identification [19], and pupil center identification [21,22,23]. The latest image segmentation models are variants of encoder-decoder architectures such as U-Net [24] and fully convolutional networks (FCN) [25].

At present, all deep learning (DL) models, such as those involving face recognition [26,27,28,29,30,31], need a huge volume of data to enhance the accuracy of the model during training. Therefore, collecting a large amount of diversified training data is very important for training robust and accurate deep neural networks. The lack of training data negatively affects the performance of the training process. In the iris databases, the CASIA-Iris-Thousand database published by the Chinese Academy of Sciences is recently the biggest available database [32], but even for this dataset, it includes only 20,000 images and cannot be called as large scale in the field of deep learning. There is a great need for a database bigger than CASIA-Iris-Thousand to develop deep-learning-based algorithms for iris segmentation and recognition. However, obtaining a huge iris database like CASIA-Iris-Thousand already requires extensive human labor costs. 

There is a recent trend in DL using the Self-Supervised Learning (SSL) framework to train a model. Self-Supervision methods have shown great potential in various research tasks ranging from computer vision to robotics [33,34,35,36]. SSL is different from supervised learning, in which we need ground truths (labels for data) for every image in the dataset. To obtain high-quality labeled data is an exhaustive and time-consuming task, especially for complicated tasks, for instance, object detection and semantic segmentation, which require highly precise annotations. On the other hand, with SSL, we only need label information for just a small amount of data in the whole dataset. The motive behind SSL is to learn valuable representations of input data from unlabeled data without relying on human annotations [37]. In this work, we intend to propose a novel framework of SSL to apply in iris segmentation networks. To our knowledge, this seminal study has applied the SSL concept to iris segmentation network training. 

Our study essentially is based on Generative Adversarial Networks (GAN) [38,39,40], which gives a powerful framework to learn to produce examples from a provided distribution. The GAN framework consists of a generator model for producing new reasonable synthetic images and a discriminator model that classifies the images as authentic (from the data set) or fake (generated). The two models are trained simultaneously in an adversarial process where the goal of the generative model is to generate a sample so that the discriminative model cannot distinguish whether it is a generated sample or an original one. The goal of the discriminative model is to successfully find the actual image without being confused by the image generated by the generative model [41]. The Pix2Pix method proposed by Isola et al. [42] is a GAN model designed for general purpose image-to-image translation. The Pix2Pix model is a kind of conditional GANs (cGANs) [43] in which the generation of the output image depends on the input image. The discriminator produces both the source image and the target image and needs to learn whether the target is a reasonable transformation of the source image. The Pix2Pix GAN has proven in a series of image-to-image translation tasks, such as converting maps into satellite pictures, black and white pictures into colors, and product sketches into product pictures.

In terms of the works for semantic segmentation for iris images, both feature-based machine learning techniques and the recently popular DL techniques require a certain number of iris images with manually marked pupil center and radius, iris center, radius, and available iris regions to advance the performance of the algorithm. In this paper, we use our proposed Pix2Pix conditional generative adversarial network to generate an iris database with pre-conditioned information such as the exact location and shape of the pupil center and radius, iris center and radius, and available iris region to improve the performance of deep learning-based algorithms. We collected two challenging datasets for training and evaluation of the proposed model: CASIA-Iris-Thousand and Iris Challenge Evaluation (ICE). Each iris image is manually annotated with the pupil center and the inner and outer boundaries of the iris as an additional source of ground truth. For the iris mask, we label the eyelashes and reflective points in eye images. For the periocular masks, the region of the eye for each image is manually denoted. These datasets comprise several types of noise, for example, blur, off-axis, occlusion, and specular reflection. One goal of this work is to train a Pix2Pix cGAN model in order to generate iris images given pre-conditioned periocular masks and iris masks. After such cGAN model is well trained, it is able to generate (synthesize) as many iris images as we want, and these iris images, which come with pre-conditioned periocular masks and iris masks, can be used to train the semantic iris segmentation model. Because the size of the training set can be as big as we want, the precision of the semantic iris segmentation model can be greatly enhanced compared to the traditional training procedure for deep models. To evaluate whether the generated iris image is useful, we further use the real iris images to test the segmentation model.

The main contribution of this paper is summarized as follows:

1. We introduce an improved version of the Pix2Pix-based conditional adversarial generative (cGAN) model, which can serve to generate a vast amount of iris images with pre-defined iris masks and periocular masks. The size of the generated iris database is unlimited and can be as big as we want.
2. Our approach can produce high-quality and diversified iris images, not only increasing the amount of the data.
3. The creation of the pre-defined iris masks and periocular masks in our framework is fully parameterized. Therefore, they can be automatically generated. It means the generation process of iris images, iris masks, and periocular masks can be fully automated in the proposed framework, and no human intervention is required. In this proposed framework, since only a small number of images that require annotation are needed, it can be seen as a self-supervised learning framework.
4. The proposed framework can be easily extended to image segmentation network training for any specific target object, as long as the shape of the target object can be parameterized. Therefore, the proposed framework has high generalization ability.

The rest of the paper is organized into the following sections. Section 2 describes the related work. In Section 3, the proposed method is presented. Experimental results and discussion are presented in Section 4. Finally, Section 5 draws the conclusion and directions of future works.

## 2. Related Works

Generally, in most inherited iris segmentation approaches, the inner and outer iris boundaries are detected first, later by further positioning the upper and lower eyelids, a refined iris mask is taken (excluding any overlapping occlusions of shadows, glasses, eyelashes, or reflections). It means that iris localization appears first, followed by narrow-defined iris segmentation [44]. In general, segmentation approaches can be divided into two main classes: boundary-based and pixel-based. The boundary-based strategy mainly determines the pupil, edge, and eyelid boundary to quarantine the iris texture area. In contrast, the pixel-based approach immediately discriminates iris pixels from non-iris pixels based on pixel-level features description [2]. 

### 2.1. Boundary-Based Segmentation Technique

For boundary-based approaches, Daugman’s integrodifferential operator [11] and Wilde’s circular Hough transform [45] are the two widely used baseline algorithms. The most significant and primary supposition made by these two approaches is that the pupil and edge borders are circular shapes. The integrodifferential operator quests for the highest variation in intensity in the parameter range ordinarily corresponding to the edge of the pupil and the iris, while the Hough transform finds the best circle parameters in the binary edge image through a voting method. Although these approaches have obtained immeasurable segmentation success in iris images taken in self-restrained conditions, these are time-wasting and not appropriate for degenerate ocular iris images. Several techniques have been introduced to address these challenges and to enhance the robustness and effectiveness of bounding-based iris segmentation techniques, such as noise elimination [46,47], poor iris location [48,49], and various models’ selection [50].

### 2.2. Pixel-Based Segmentation Technique

On the contrary, to directly identify the iris and non-iris regions, there are some pixel-based iris segmentation methods. In most cases, they use the low-level visual description of each pixel (e.g., intensity and hue) to separate the pixels of interest from the background image. The well-known pixel-level methods like Graph Cut [51,52] can pre-process images, while conventional classification techniques such as Support Vector Machine SVM [53] can classify iris pixels from non-iris pixels. Based on prior knowledge, modern boundary-based and pixel-based approaches require a great deal of pre-processing and post-processing. The iris segmentation method based on deep learning can directly estimate the iris mask and automatically learn the best features but utilizes more high-level semantic features. They are end-to-end prediction models in which classifiers and features are jointly optimized, and no additional pre-processing and post-processing are required. Li et al. [54] proposed an iris segmentation method based on deep learning, which combines edge-based and learning-based algorithms. Liu et al. [16] introduced a pixel-based iris segmentation model to automatically learn iris pixels. Later, researchers utilized existing [55,56,57,58], customized [18,59], and fully connected networks (FCN) models for iris segmentation and gained the best segmentation accuracy on several iris datasets. Li et al. [1], Lian et al. [58], Lozej et al. [60], Wu and Zhao [61], and Zhang et al. [62], scholars employed alternatives of U-Net [24] for iris segmentation.

### 2.3. Semantic Segmentation Technique

The semantic segmentation task can be examined as a pixel-by-pixel image classification process, where every pixel within the image is assigned an object class. Long et al. [25] first presented a Fully Convolutional Network (FCN) for semantic segmentation in 2005. After that, many FCN-based semantic segmentation methods have been introduced, for example DeepLab set [63,64], U-Net [24], and PSPNet [65], to enhance the capability of semantic segmentation. U-Net [36] is an extensively employed network for medical image segmentation examination. It is further enlarged to 3D U-Net [66], TernausNet [67] and U-Net++ [68], and has good performance on general image segmentation tasks. The FCN-based method takes the entire image as input and generates a probability distribution map using a sequence of convolutional layers without including fully connected layers. In this model, everything is fully automated, no manual effort is required, and it takes advantage of the most advanced technology currently available. Iris segmentation can be perceived as a particular binary semantic segmentation problem. Therefore, several FCN-based segmentation techniques can be directly applied to iris images, such as [16,17,18,20].

### 2.4. Generative Adversarial Network (GAN)

Regarding the latest development in the research of GAN, several approaches have been introduced to generate images. In [69] propose a convolutional GAN model for image generation, which has excellent performance to fully connected networks. In 2018, Minaee and Abdolrashidi [70] presented an iris image generation framework named Iris-GAN was proposed, which uses a simple deep convolution GAN model to generate realistic iris images that are indistinguishable from the actual iris image. Mirza and Osindero [43] introduced a conditional GAN model, which is able to generate images conditioned on class annotations. Zhu et al. [71] proposed an image-to-image conversion model based on a cyclic consistent GAN model that learns to map a given image distribution to a target domain. Ledig et al. [72] proposed a GAN-based image super-resolution approach, which attempts to produce a high-resolution (HR) variant of images that seem related to the target HR. The concept of adversarial training has also been implemented in the autoencoder framework to present an unsupervised feature learning approach [73]. In 2019, a model named RaSGAN [74] was proposed, which accentuates the relativistic of the GAN to be further generalized by updating its loss function. Several other works extend the GAN model in different approaches.

## 3. Proposed Method

### 3.1. Framework Overview

**Figure 1:**
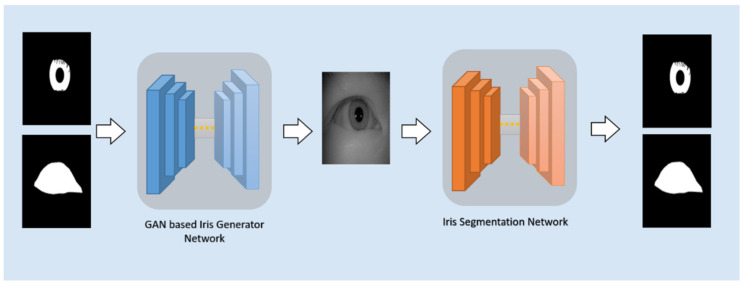
An overview of our framework.

Inspired by the success of Pix2Pix on learning to map the input image to the output image, the overall design of our framework is depicted in Figure 1. Our proposed framework contains two networks: the iris image generation network and the iris segmentation network. Our goal is to train a robust iris segmentation network, which is able to deal with all kinds of iris images, including irises with different shapes, rotation angles, different sizes of pupil, among other aspects. To achieve this, first, we train the iris image generation network to generate immense diversified iris images, given a set of diversified iris masks and periocular masks, which can be fully parameterized. Then, the generated images are applied as a training set to train the iris segmentation network. 

### 3.2. Iris Image Generation Network

**Figure 2:**
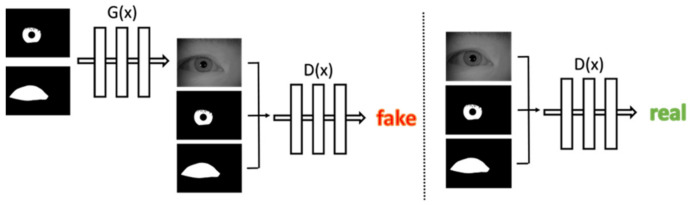
Proposed method training architecture.

The initial Generative Adversarial Network (GAN) is to learn a mapping from random noise to the output image. On the contrary, in this study, we propose a network which is able to learn a conditioned mapping from pre-defined iris and periocular masks to the real iris images, as shown in Figure 2. The network consists of two competing networks. The generator G, which attempts to generate the most authentic appearance of iris images and the discriminator D, which learns to distinguish the real and synthetic iris images. To train the network, first, we use the iris mask and periocular mask combined as a set of two-channel images to be the input, and generator G generates a realistic iris image to deceive the discriminator D. Then, we merged the iris mask, periocular mask, and iris image into a set of three-channel images and let the discriminator D determine whether this set of images is real (iris images from real-world) or false (iris images generated by G). 

**Figure 3:**
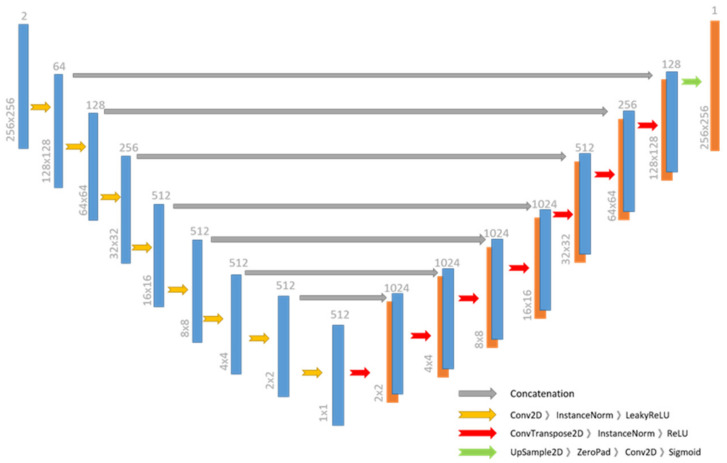
The generator architecture in the proposed method.

For the generator network G, we propose a network structure modified from U-Net architecture with two main parts: the encoder, which learns the feature embedding from the input images and the decoder, which hallucinates the output images based on the given embedding. The overall structure of the generator network is shown in Figure 3. The encoder part consists of eight convolutional layers followed by instance-normalization and LeakyReLU as the activation function. The input is an image pair spatial resolution 256 × 256, the kernel size 4 × 4, and the stride is 2. The decoder part adopts 8 transpose convolutional layers followed by instance normalization and ReLU as the non-linearity to perform the process of image hallucination. The sigmoid function is applied to the last convolution layer. In order to evade the loss of feature information, we adopted skip connection to every convolutional layer, which incorporates the feature maps on the encoder part with the decoder part. At the same time, dropout was added with a probability of 0.5 to the first five layers of the decoder part. 

**Figure 4:**
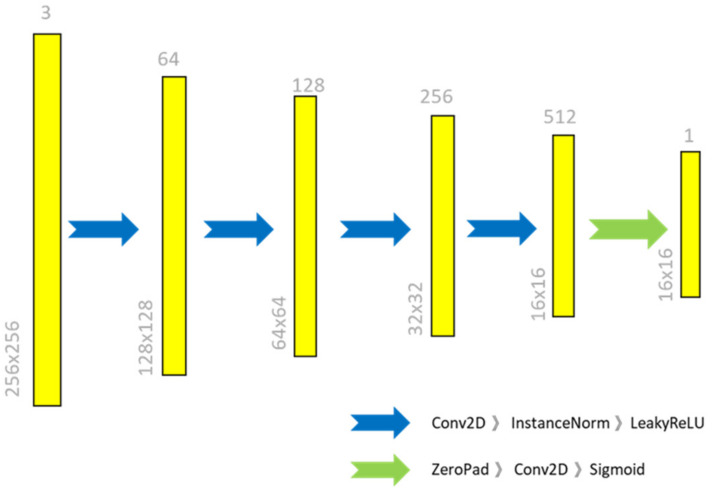
The discriminator architecture in the proposed method.

The discriminator network D consists of five convolutional layers with a kernel size of 4 × 4 and strides of 2, followed by instance-normalization and leaky ReLU as the activation function. After the fourth convolution layer, the size of the feature maps is reduced to 16 × 16. The discriminator network architecture is shown in Figure 4.

### 3.3. Objective Function 

The loss function of our network is defined as Equation (1).

(1) $$L_{cGAN}\left(G,D\right)={Ε}_{\left(x,y\right)\sim P_{data}}\left[Log\left(D\left(x,y\right)\right)\right]+{Ε}_{x\sim P_{data}}\left[Log(1-D\left(x,G\left(x\right)\right)\right],$$

where $E\left(\cdot \right)$ represents the expectation operator, $G\left(\cdot \right)$ represents output from the generator network, $D\left(\cdot \right)$ represents the output from the discriminator network, x represents the mask input to the network, y is the real iris image corresponding to x, $P_{data}$ is the distribution of real data, $\left(x,y\right)$ belongs to $P_{data}$.

The objective function during the optimization process can be expressed by the Equation (2):

(2) $$G^{*}=arg\;\min_{G}max_{D}L_{cGAN}\left(G,D\right).$$

Earlier methods have discovered that it is advantageous to join the GAN goal with a more popular loss, for example, *L*2 distance [75]. The discriminator retains its role, but the generator is modified not just to mislead the discriminator further to approximate the ground-truth output in an *L*2 sense. Furthermore, we investigate the alternative by applying *L*1 distance instead of *L*2 because *L*1 tends to cause scarcer obscure. It is described in Equation (3):

(3) $$L_{L1}\left(G\right)=E_{\left(x,y\right)\sim P_{data}}\left[\left\|y-G\left(x\right)\right\|\right].$$

Therefore, our final objective function is expressed by Equation (4), where λ is the weight for *L*1 norm (λ=100):

(4) $$G^{*}=arg{\;\min}_{G}max_{D}L_{GAN}\left(G,D\right)+\lambda L_{L1}\left(G\right).$$

### 3.4. Iris Segmentation Network

For the training of the image segmentation, we employed the same architecture as the generator from the iris image generation network and modified the input channel to one. Since most iris biometrics operates on grayscale images, the input to the network is defined to be a grayscale image with spatial resolution 256 × 256. The detailed architecture is shown in Figure 3. The goal of the semantic segmentation network can be described as classifying each pixel on the input image into different categories. Instead of utilizing the commonly used cross-entropy loss, we adopted the loss function of FCN as expressed by Equation (5) to add up the loss function at all pixel positions.

(5) $$L\left(P,Q\right)=-\overset{w}{\underset{i=1}{\sum }}\overset{h}{\underset{j=1}{\sum }}\overset{n}{\underset{k=1}{\sum }}Q_{k}\left(i,j\right)\log P_{k}\left(i,j\right),$$

where *w*, *h* represents the width and height in the image, respectively; *n* represents the total number of categories to be classified; $P_{k}\left(i,j\right)$ represents the probability value of the *k*th category for the pixel located on the position $\left(i,\;j\right)$; $Q_{k}\left(x,y\right)$ is the label of the *k*th category on the *x*-axis at position *i* and the y-axis at position *j*, which is equivalent to adding up the cross-entropy loss on each pixel to get the final overall loss value.

In order to optimize this semantic segmentation, we used mini-batch SGD and Adam optimizer. The learning rate is set to 1e^−5^; the momentum is set to β_1 = 0.5, β_2 = 0.99; batch size is set to 64, and Gaussian distribution is used to initialize the parameters in the network. A total of 10 epochs are iterated. 

### 3.5. Automatic Mask Generator

**Figure 5:**
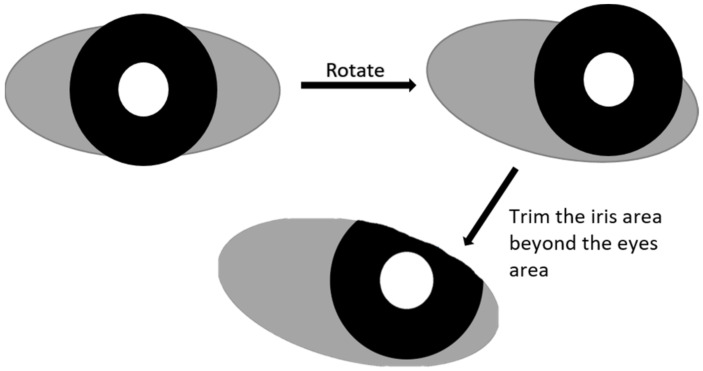
The process of automatic mask generation.

**Figure 6:**
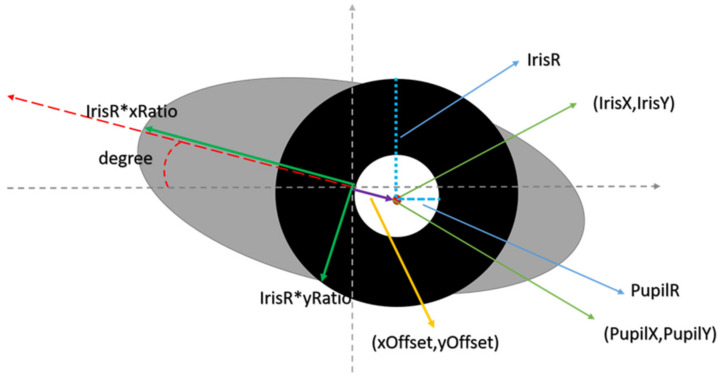
A pictorial explanation to determine the 11 parameters to generate the masks.

In response to the fact that there are not many manually labeled ground-truth masks for the test data, we designed a process to generate the iris mask and the periocular mask as the input of the neural network to facilitate the use of automated methods to generate an unlimited amount of data. As shown in Figure 5, in the first step, we assumed that the iris mask is composed of two nearly concentric circles, one large and one small, and the periocular mask is composed of an ellipse. To diversify the appearance of the output images of the network, we parameterized each of these basic components (shapes) so that all the important factors of the eye (e.g., the position, size, and rotational angles) can be specified by random parameters. In this way, the output images of the proposed network will have a random appearance, and it mimics the random distribution of the eye images, which can be collected in practical situations. As shown in Figure 6, we define the following parameters:

1. PupilX: the *X*-axis coordinate of the pupil center in the iris image
2. PupilY: the *Y*-axis coordinate of the pupil center in the iris image.
3. PupilR: the radius of the pupil in the iris image.
4. IrisX: the *X*-axis coordinate of the iris center in the iris image.
5. IrisY: the *Y*-axis coordinate of the iris center in the iris image.
6. IrisR: the radius of the iris in the iris image.
7. (xOffset, yOffset): a set of vectors representing the displacement of the centers of the eye and the iris.
8. xRatio: we use the shape of the ellipse to approximate the shape of eyes. An ellipse can be described by its center position, semi-major and semi-minor axis. The center position is parametrized by (xOffset, yOffset). xRatio is the value computed from the semi-major axis length divided by the iris radius.
9. yRatio: the value computed from the semi-minor axis length divided by the iris radius.
10. Degree: the angle of rotation of the ellipse.

To calculate the range of the 11 parameters, we performed statistical analysis on the ground truth information in the CASIA-Iris-Thousand dataset of training data to observe the parameter distribution and calculate the mean and standard deviation, as the parameter range. Since the size of the training images is 640 × 480, the range of X and Y coordinate value are [0, 640], and [0, 480], respectively. Table 1 shows the statistics of the 11 parameters.

**Figure 7:**
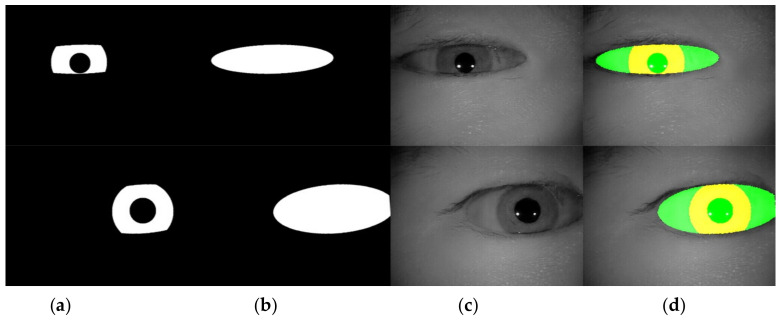
A pictorial example of generated masks for iris images. (**a**) The generated iris mask; (**b**) the generated periocular mask; (**c**) the generated iris image; (**d**) the overlay of iris mask, periocular mask and the generated image. It shows that the generated masks perfectly fit the iris image.

Based on Table 1, the value range for pupilR and irisR are set to [20, 60] and [70, 120], respectively. Because the value of xRatio and yRatio cannot be zero (otherwise the ellipse of the periocular mask will disappear), the xRatio is set to [1.3, 2.5] and the yRatio is set to [0.4, 1.1]. Besides, if the value of xOffset and yOffset are too large, the iris mask will be eliminated. A good choice is to set them to be smaller than the half distance between the long and short axis of the ellipse. Thus, the value of xOffset and yOffset are set to [−xRatio*irisR/2, +xRatio*irisR/2] and [−yRatio*irisR/2, +yRatio*irisR/2], respectively. Lastly, we set the value of degree to be within [−15, +15]. The example result of generated masks is shown in Figure 7.

## 4. Experimental Results and Discussion

### 4.1. Experimental Details

To train the iris image generation network, we initially pre-process the data by randomly cropping an image of size 608 × 456 from the original image (size 640 × 480), which will make the iris size larger in disguise and increase the generation network’s ability to generate targets with a larger radius. In addition, the data of the left eye and the right eye can be mutually augmented through the horizontal flip operation, and finally, the image size is down-sampled to 256 × 256 to match the network input. 

The proposed method is trained and tested using PyTorch deep learning framework. We performed our experiments on a machine with an NVIDIA 1080Ti GPU and 11 GB of memory, mini-batch SGD, and Adam optimizer with the learning rate 1e^−4^, and the momentum β_1_ = 0.5, β_2_ = 0.99. A Gaussian distribution is applied to initialize the parameters in the network with a batch size of 64. We performed the experiments for non-glasses and with-glasses data separately.

### 4.2. Iris Databases and Data Augmentation

This experiment used the CASIA-Iris-Thousands database [76] as well as the ICE iris database [77]. The Institute of Automation at the Chinese Academy of Sciences established the CASIA-Iris-Thousands. This database contains 20,000 images spanning a wide range of subjects. There are the same number of images in each subject’s right and left eyes. The image resolution for this database is 640 × 480. The total number of images of with-glasses and without-glasses are 5338 and 14,662, respectively. The National Institute of Standards and Technology (NIST) conducts and manages the ICE database, which contains 2953 images covering 124 subjects.

In order to improve the generalization capability of the iris generation model, we performed data augmentation on the iris datasets used for experiments. We applied the following methods for data augmentation.

1. Randomly flip horizontally with the probability of 0.5.
2. Randomly crop the image with resolution 432 × 576.
3. Resize the image to a resolution of 256 × 256.

### 4.3. Performance Evaluation

To assess the performance of the proposed method, we use evaluation metrics, which were commonly used in image segmentation works, as described below.

1. mIoU (mean Intersection over Union) is a commonly used metric in semantic segmentation, which is the average ratio of the intersection and union of the two sets of real and predicted values. The values of IoU are limited to the [0, 1] interval, with 1 representing the accurate results (100% accuracy), while 0 indicates 0% accuracy.
2. PA (Pixel Accuracy) and mPA (mean Pixel Accuracy) are the percentage of correctly marked pixels to the total pixels and the average over all classes. The value of mPA is in the range of [0, 1]. The closer the value is to one, the higher the accuracy of segmentation is.
3. Frequency Weighted Intersection over Union (FWIoU) is a metric to compensate the impact from the class imbalances issues, which is calculated using Equation (6). The $p_{ij}$ represents the pixels that belong to the $i^{th}$ category but are predicted to be the $j^{th}$ category,$\;p_{ii}$ represents the true positive value, and $p_{ji}$ represents the false positive value.
  (6) $$\mathrm{FWIoU}=\frac{1}{\sum_{i=0}^{k}\sum_{j=0}^{k}p_{ij}}\sum_{i=0}^{k}\frac{p_{ii}}{\sum_{j=0}^{k}p_{ij}+\sum_{j=0}^{k}p_{ji}-p_{ii}}.$$

### 4.4. Experimental Results and Analysis 

Since there is no objective evaluation method to examine whether the generated image is true, and our goal is to improve the deep learning algorithm for iris segmentation task by hallucinating training data, we assess the performance of generated images by analyzing the segmentation accuracy of the down-stream segmentation networks. In our experiment, 5000 iris images from CASIA-Iris-Thousands are randomly chosen as the initial training set and another 5000 are randomly chosen as testing set. With the proposed iris image generation network, we generated a lot of iris images which serve as additional training data for training the segmentation network. A detailed description of the three sets of training data is given as follows:

1. The 5000 iris images randomly chosen from CASIA-Iris-Thousands.
2. The 15,000 iris images consisted of training set 1 and 10,000 additional iris images produced from the proposed iris image generation network.
3. The 25,000 iris images consisted of training set 2 and 10,000 additional iris images generated from the proposed iris image generation network.

**Figure 8:**
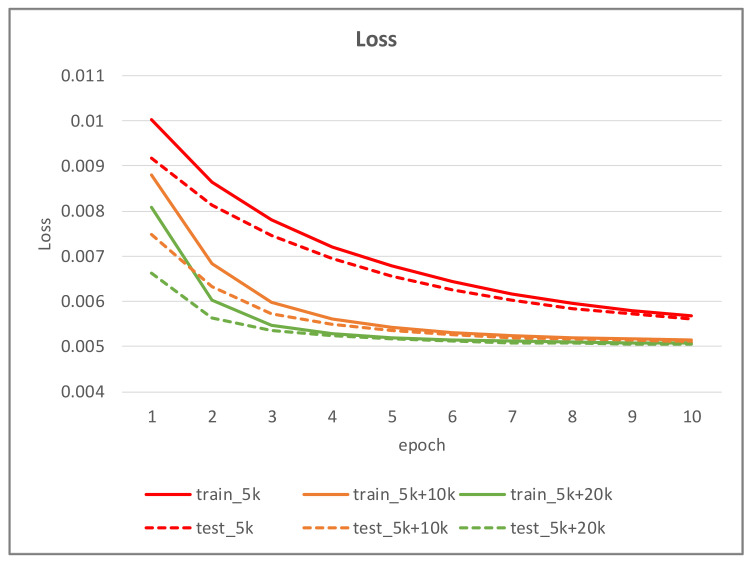
The loss curve of the proposed method on training data and testing data.

**Figure 9:**
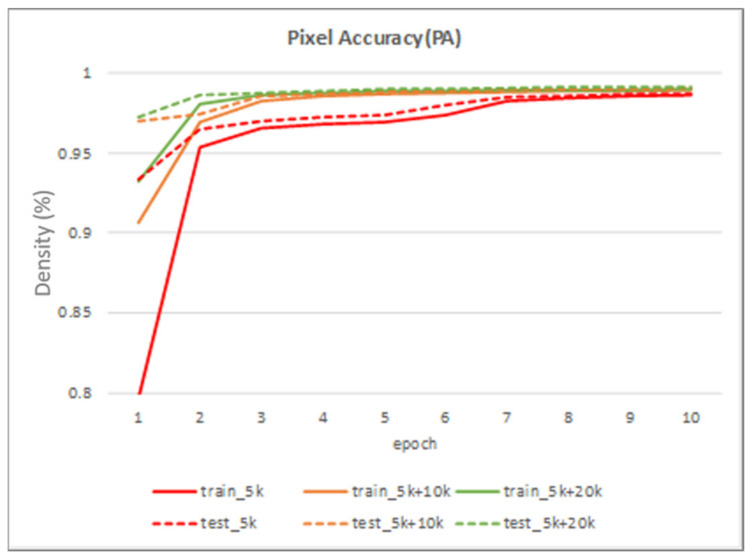
The pixel accuracy curve on training data and testing data.

**Figure 10:**
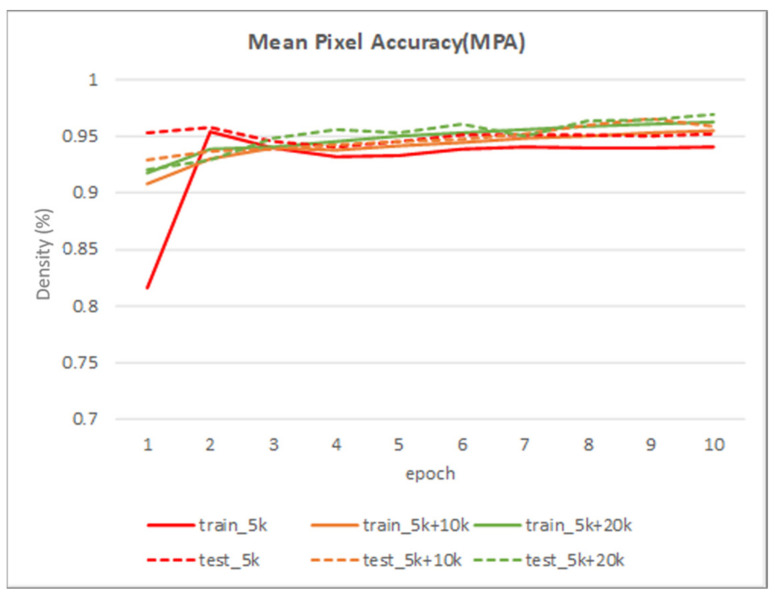
The mean pixel accuracy curve on training data and testing data.

**Figure 11:**
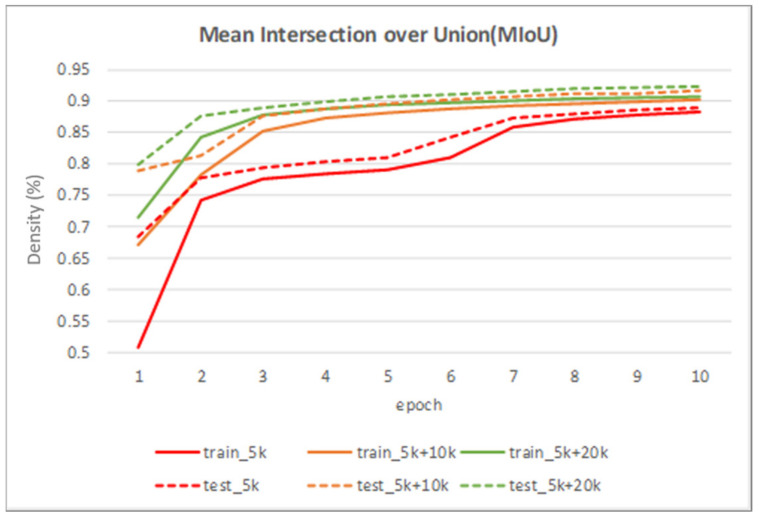
The mean intersection over union curve on training data and testing data.

**Figure 12:**
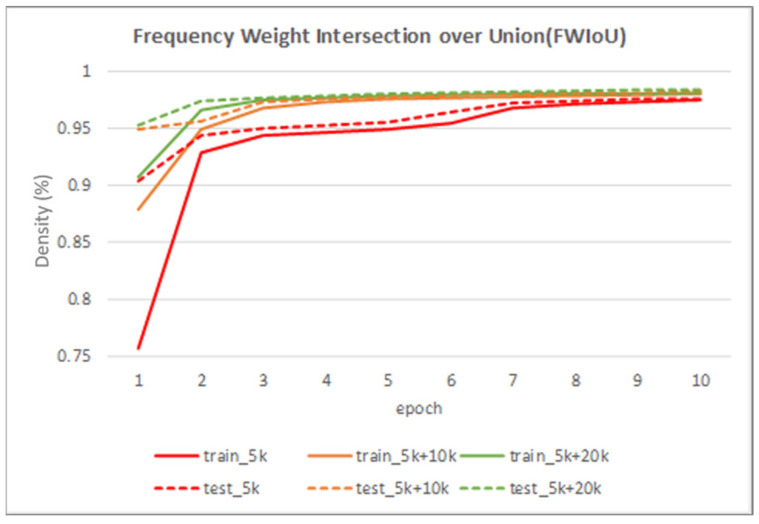
The frequency weight intersection over union curve on training data and testing data.

**Figure 13:**
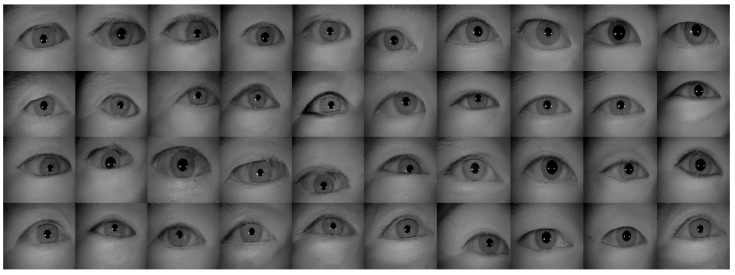
The examples of generated images (trained with CASIA-Iris-Thousands, without glasses) using the proposed iris image generation network.

**Figure 14:**
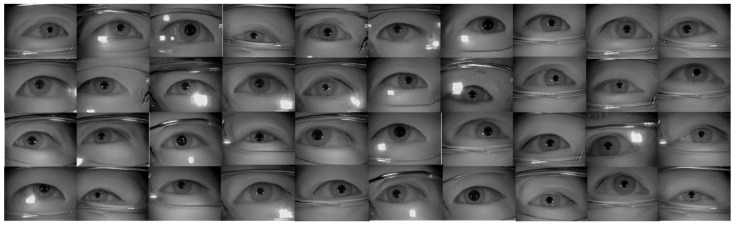
The examples of generated images (trained with CASIA-Iris-Thousands, with glasses) using the proposed iris image generation network.

Table 2 shows the performance of the segmentation model U-Net [24] trained with the three training datasets. As can be seen, the performance of iris segmentation increases when the number of training data increases. The results on mIoU show a large margin when the number of training data grows from 5000 images to 25,000 images (from 88.9% to 92.4%). For the results on other evaluation metrics, the number of training data clearly affects the performance of the segmentation model. This means the proposed method successfully generates high-quality and realistic images which are applicable to the iris segmentation task. Figure 8 shows the trend during the training on three different sets of data. Figure 9, Figure 10, Figure 11 and Figure 12 show the pixels accuracy curves, mean pixels accuracy curves, mean IoU curve, and frequency weight IoU curve for different sets of training data, respectively. From those figures, we can see that the optimization of the segmentation model converges faster with the increasing number of epochs in the training process. The curves are relatively stable without oscillations, indicating that the model is trained well. By comparing the loss and accuracy between the training and testing, we can see that there is no overfitting phenomenon. The examplar images from the iris image generation network are shown in Figure 13 and Figure 14.

### 4.5. Experimental Results and Analysis on ICE Database

**Figure 15:**
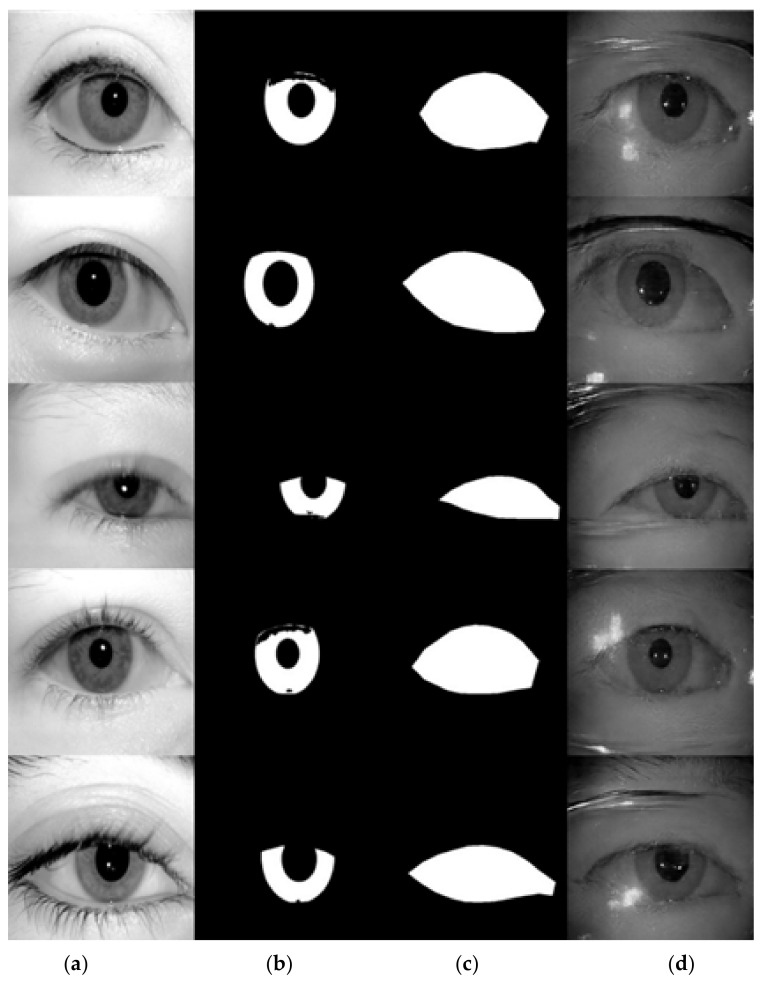
The examples of generated images (trained with ICE dataset, with glasses). (**a**) original iris image; (**b**) ground-truth label for iris; (**c**) ground-truth label for periocular region; (**d**) generated images using (**b**,**c**) as the conditional inputs to the proposed image generation network.

**Figure 16:**
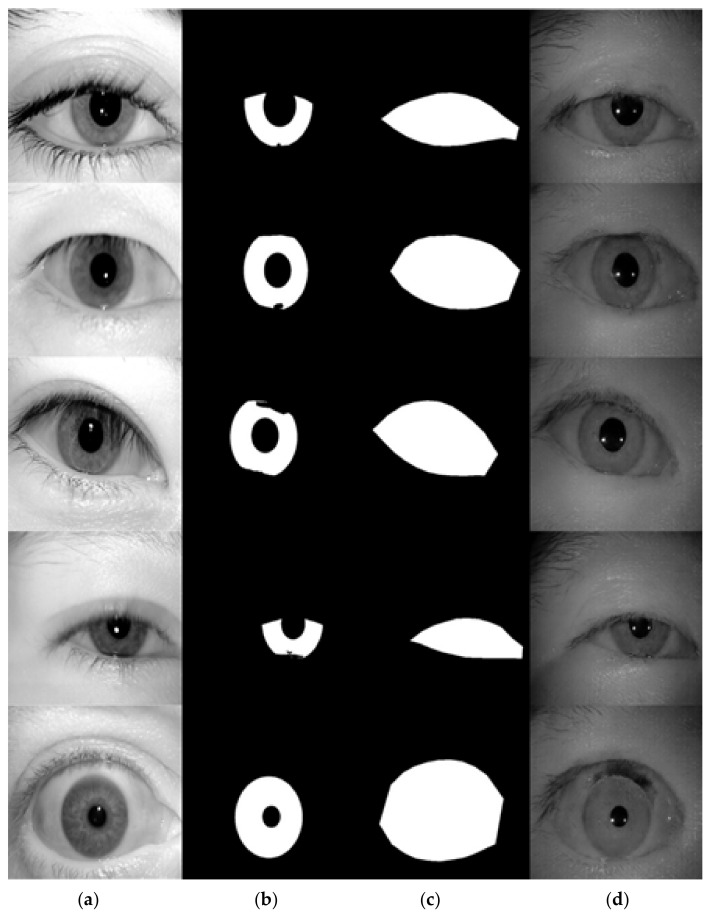
The examples of generated images (trained with ICE dataset, without glasses). (**a**) original iris image; (**b**) ground-truth label for iris; (**c**) ground-truth label for periocular region; (**d**) generated images using (**b**,**c**) as the conditional inputs to the proposed image generation network.

**Figure 17:**
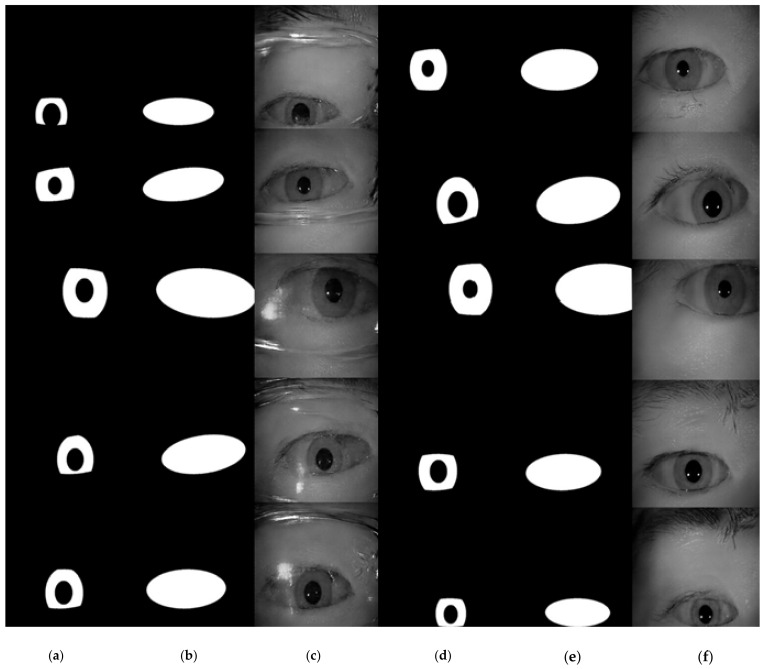
The examples of generated images (trained with ICE dataset, with and without glasses). (**a**,**d**) generated iris mask; (**b**,**e**) generated periocular mask; (**c**) generated image with glasses; (**f**) generated image without glasses.

In our experiment on the ICE database, we used two different sets of masks as the input to the network, one is to use the ICE database ground-truth label as the conditional input to the network, and the other is to use the mask generated by the proposed algorithm mentioned in Section 3.5 as the conditional input for iris image generation network. The sample of generated images using the ground-truth label for images with glasses and without glasses are manifested in Figure 15 and Figure 16, accordingly. The proposed framework clearly generated realistic images for irises in both with-glasses and without-glasses cases. The following generated images using the algorithm in Section 3.5 for both cases can be seen in Figure 17. As can be seen, the proposed mask generation algorithm successfully helps to generate very realistic images. Therefore, the conditional mask input to the proposed network can be fully automated and so is the whole process of the iris image generation. With the proposed image generation network, the amount of training datasets can be increased to any predefined size, which greatly enlarges the training resources needed for semantic segmentation. 

### 4.6. Comparison with Existing Segmentation Algorithms

**Figure 18:**
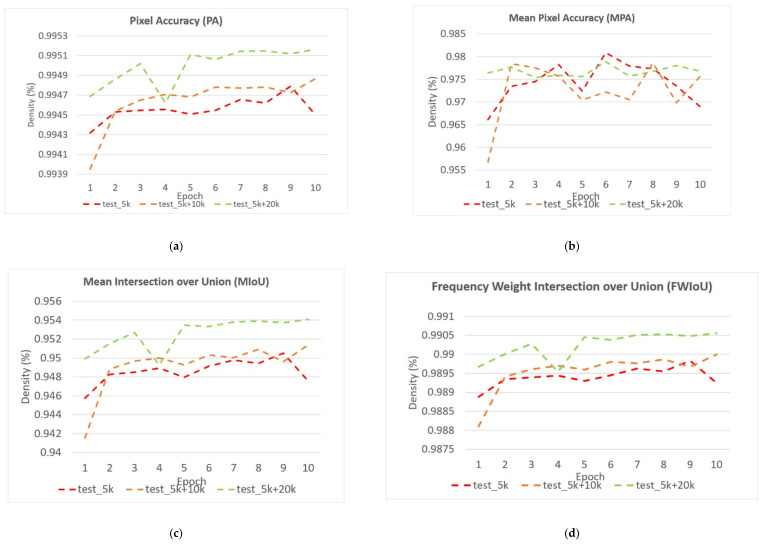
The performance of FCN network on customized dataset based on the value of: (**a**) Pixel Accuracy (PA); (**b**) Mean Pixel Accuracy (MPA); (**c**) Mean Intersection over Union (MIoU); (**d**) Frequency Weight IoU (FWIoU).

**Figure 19:**
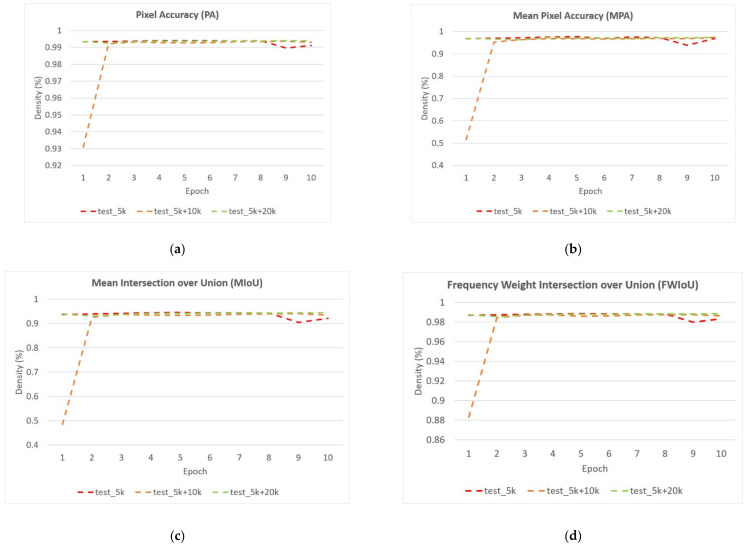
The performance of Deeplab network on customized dataset based on the value of: (**a**) Pixel Accuracy (PA); (**b**) Mean Pixel Accuracy (MPA); (**c**) Mean Intersection over Union (MIoU); (**d**) Frequency Weight IoU (FWIoU).

Towards the objective of examining the performance of the introduced method, we employed the current state-of-the-art (SOTA) segmentation model U-Net [24], FCN [25], and Deeplab [64] as the down-stream semantic segmentation networks. In this experiment, we utilized FCN network with VGG-16 backbone and Deeplab with ResNet101 backbone for the purpose of comparison. The models were trained and tested under the same configuration with the learning rate, batch size, and epoch equal to 0.001, 64, and 10, respectively. The results were evaluated on evaluation metrics from Section 4.3 shown in Table 3. As can be seen, the performance of each SOTA model trained with generated data achieves promising results. It shows that the generated images by the proposed network can improve the performance and are applicable for training the semantic segmentation task. Figure 18 and Figure 19 show the detailed performance of FCN and Deeplab networks evaluated on various metrics. 

### 4.7. Analysis on Generated Image Quality

In order to measure the diversity and quality of the proposed model, we calculated the Frechet Inception Distance (FID) [78] on the generated iris images. The FID compares the statistics of the generated images to the real images:

(7) $$\mathrm{FID}=\|\mu_{r}-\mu_{g}\|{}^{2}+Tr\left(\sum_{r}+\sum_{g}-2\sqrt{\sum_{r}\sum_{g}}\right),$$

where $\mu_{r}$ and $\sum_{r}$ represents the statistics of the real image distribution, $\mu_{g}$ and $\sum_{g}$ represents the statistics of the generated image distribution, and Tr is the trace of the covariance matrix $\left(\sum_{r}+\sum_{g}-2\sqrt{\sum_{r}\sum_{g}}\right)$. The FID score is measured as the distance between two distributions; the lower the score, the higher similarity between the generated images and real images. In our experiment, we compared the quality of the generated images by our proposed network with the prior works from Minaee and Abdolrashidi [70] and Yadav et al. [74] on the CASIA-Iris-Thousands database under the same configuration as mentioned in Section 4.1. The FID scores of both networks are shown in Table 4. 

**Figure 20:**
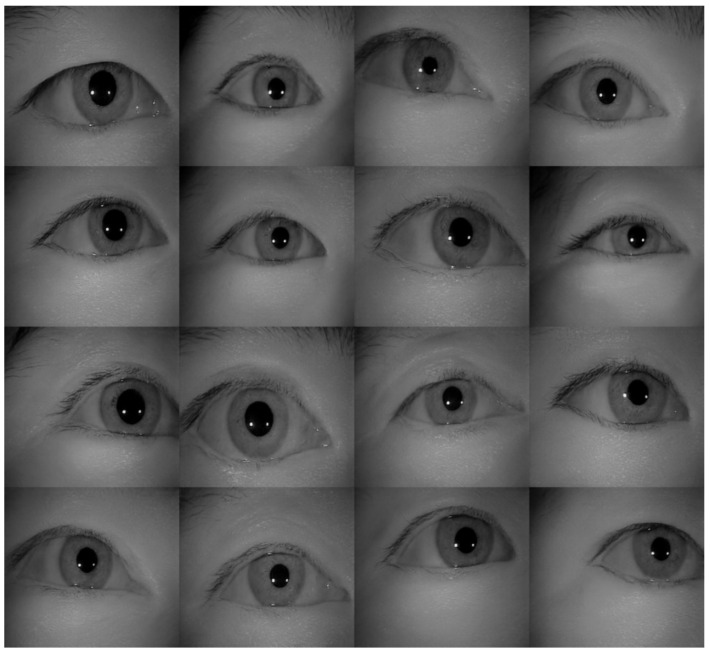
The examples of generated images by the proposed network.

**Figure 21:**
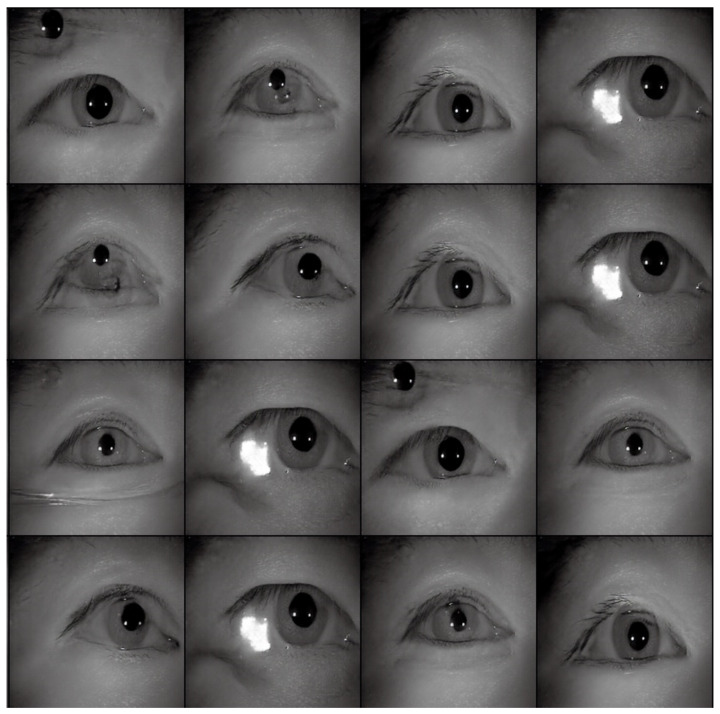
The example of generated images by Minaee and Abdolrashidi [70].

**Figure 22:**
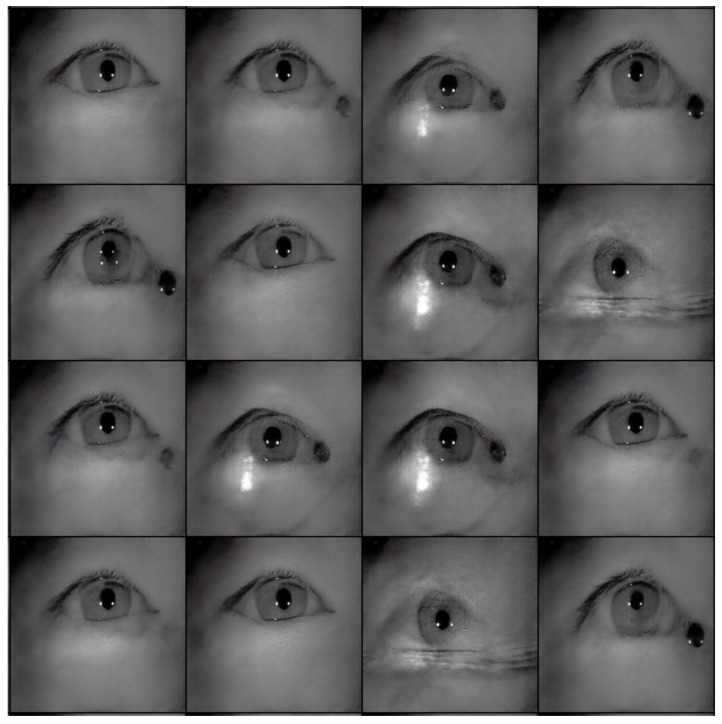
The example of generated images by Yadav et al. [74].

As shown in Table 4, the proposed network achieved a lower FID score compared to the prior works. Hence, we can conclude that the generated images by the proposed method closely resemble the real images and are applicable to train the iris segmentation network. Moreover, compared to the prior works, the strength of the proposed network for the down-stream tasks are listed in Table 5. The sample of generated images by the proposed method and the prior works by Minaee and Abdolrashidi [70] and Yadav et al. [74] are shown in Figure 20, Figure 21 and Figure 22, respectively.

In Figure 20, we can see that the proposed network can generate more realistic iris images. Compared with the generated images by the prior works in Figure 21 and Figure 22, the generated iris images by the proposed network look much more natural with high quality, which looks like genuine images extracted from the dataset. The iris images generated by the prior works showed an unnatural appearance, such as images with more than one irises, pupils outside the eye, blurry images, and even images with two eyes. From these observations, we can conclude that the proposed network outperforms the state-of-the-art, and the results are suitable for the down-stream tasks such as presentation attack, iris detection, iris segmentation …etc. 

## 5. Conclusions

We proposed a self-supervised framework overcoming the problem of data scarcity for the purpose of training an accurate segmentation network. In this paper, we proposed a Pix2Pix-based conditional generative adversarial network architecture to generate photo-realistic iris images. We utilized both iris mask and periocular mask as the condition for the proposed image generation network. The generated images are used as the additional training data to train the iris segmentation network. For the conditional input (masks of iris and eyes) of the image generation network, we proposed an analytic method based on 11 programmable parameters, which can be randomly generated. In the experiments, a few large-scale experiments are designed and executed to evaluate the performance of the proposed framework. The performance of the trained image segmentors grows linearly with the size of the overall training data, which shows the feasibility and effectiveness of the proposed framework. 

The proposed framework can be easily extended to be utilized in semantic segmentation for any specific target. By modifying the parametric mask generation algorithm in Section 3.5 to adapt to the properties of the target object, such framework can be generalized to any specific target relevant to interesting specific domains in computer vision. An example is that it can be applied to face image generation by modifying the mask generation algorithm to be a parametric method to generate the facial landmark.

For future work, as stated above, we plan to apply the proposed framework to other popular domains in computer vision such as faces, cars or street views. Another direction is to re-design the backbone of the image generation models in order to generate more details for the target object, like iris patterns. Spatial attention or channel attention models are possible choices to be considered.

## Figures and Tables

**Table 1:** The statistics of the 11 parameters used for generating the iris mask and periocular mask.

Parameter Name	Average Value	Standard Deviation	Minimum Value	Maximum Value
PupilX	363.18	48.78	137.65	383.09
PupilY	222.68	41.93	75	383.09
PupilR	35.87	8.03	16.86	70.39
IrisX	360.28	48.85	135.35	546.27
IrisY	222.28	42.11	70.17	384.45
IrisR	94.57	7.41	71.31	124.97
xOffset	−2.59	17.24	−88.11	79.44
yOffset	8.03	9.5	−29.11	51.13
xRatio	2.08	0.16	0.054	2.7
yRatio	0.76	0.1	0.04	1.14
Degree	2.24	4.07	−16.2	19.38

**Table 2:** The comparison of segmentation accuracy on the U-Net trained with different number of training images generated by the proposed image generation network.

Number of Data	PA	mPA	mIoU	FwIoU
5k (real data)	98.7216%	95.2517%	88.9159%	97.6204%
5k (real data) + 10k (generated data)	99.0971%	95.9337%	91.7642%	98.2756%
5k (real data) + 20k (generated data)	**99.1622%**	**96.9423%**	**92.4142%**	**98.4049%**

**Table 3:** The evaluation result of different algorithms on the customized dataset.

Model	Number of Data	PA	mPA	mIoU	FwIoU
U-Net [24]	5k (real data)	98.7216%	95.2517%	88.9159%	97.6204%
	5k (real data) + 10k (gen. data)	99.0971%	95.9337%	91.7642%	98.2756%
	5k (real data) + 20k (gen. data)	**99.1622%**	**96.9423%**	**92.4142%**	**98.4049%**
FCN [25]	5k (real data)	99.4497%	96.8995%	94.7542%	98.9245%
	5k (real data) + 10k (gen. data)	99.4866%	97.5631%	95.1388%	98.9994%
	5k (real data) + 20k (gen. data)	**99.5164%**	**97.6801%**	**95.4050%**	**99.0558%**
Deeplab [64]	5k (real data)	99.1217%	96.8730%	92.0923%	98.3326%
	5k (real data) + 10k (gen. data)	99.3034%	97.3882%	93.4433%	98.6379%
	5k (real data) + 20k (gen. data)	**99.4051%**	**97.3951%**	**94.4271%**	**98.8472%**

**Table 4:** Frechet Inception Distance (FID) score of CASIA-Iris-Thousands database.

Network	FID
Proposed	60.25
Minaee and Abdolrashidi [70]	112.70
Yadav et al. [74]	110.56

**Table 5:** The strength and weakness of the proposed method compared to the prior work on down-stream tasks.

Properties	Proposed	Minaee and Abdolrashidi [70]	Yadav et al. [74]
Is it suitable for Presentation Attack Detection task?	Yes	Yes	Yes
Is it suitable for iris segmentation task?	Yes	No	No
Can it generate iris images based on specified iris center and radius?	Yes	No	No
Can it generate iris images based on specified pupil center and radius?	Yes	No	No
Can it generate iris images at any specified coordinate on the whole image?	Yes	No	No
Can it generate iris images with or without glasses according to the prior specification?	Yes	No	No
Can it generate iris images based on different eyelid shape?	Yes	No	No

## Data Availability

In this study, we use a public iris dataset, CASIA-v4, that can be found here: http://www.cbsr.ia.ac.cn/china/Iris%20Databases%20CH.asp (accessed on 20 July 2021).
